# Predictors of abstinence among smokers recruited actively to quitline support

**DOI:** 10.1111/j.1360-0443.2012.03998.x

**Published:** 2012-08-28

**Authors:** Flora Tzelepis, Christine L Paul, Raoul A Walsh, John Wiggers, Sarah L Duncan, Jenny Knight

**Affiliations:** 1 Priority Research Centre for Health Behaviour, University of Newcastle and Hunter Medical Research InstituteCallaghan, NSW, Australia; 2 Centre for Health Research and Psycho-oncology, Cancer Council New South Wales, University of Newcastle and Hunter Medical Research InstituteCallaghan, NSW, Australia; 3 Hunter New England Population Health, Hunter New England Local Health District, University of Newcastle and Hunter Medical Research InstituteWallsend, NSW, Australia

**Keywords:** Active recruitment, predictors, quitline, randomized controlled trial, smoking cessation, telephone counseling

## Abstract

**Aims:**

Active recruitment of smokers increases the reach of quitlines; however, some quitlines restrict proactive telephone counselling (i.e. counsellor-initiated calls) to smokers ready to quit within 30 days. Identifying characteristics associated with successful quitting by actively recruited smokers could help to distinguish those most likely to benefit from proactive telephone counselling. This study assessed the baseline characteristics of actively recruited smokers associated with prolonged abstinence at 4, 7 and 13 months and the proportion achieving prolonged abstinence that would miss out on proactive telephone counselling if such support was offered only to smokers intending to quit within 30 days at baseline.

**Design:**

Secondary analysis of a randomized controlled trial in which the baseline characteristics associated with prolonged abstinence were examined.

**Setting:**

New South Wales (NSW) community, Australia.

**Participants:**

A total of 1562 smokers recruited at random from the electronic NSW telephone directory.

**Measurements:**

Baseline socio-demographic and smoking-related characteristics associated with prolonged abstinence at 4, 7 and 13 months post-recruitment.

**Findings:**

Waiting more than an hour to smoke after waking and intention to quit within 30 days at baseline predicted five of the six prolonged abstinence measures. If proactive telephone counselling was restricted to smokers who at baseline intended to quit within 30 days, 53.8–65.9% of experimental group participants who achieved prolonged abstinence would miss out on telephone support.

**Conclusions:**

Less addicted and more motivated smokers who are actively recruited to quitline support are more likely to achieve abstinence. Most actively recruited smokers reported no intention to quit within the next 30 days, but such smokers still achieved long-term abstinence.

## Introduction

Quitline services offer proactive telephone counselling (i.e. quitline-initiated calls to smokers) and/or reactive telephone counselling (i.e. immediate counselling to smoker-initiated calls) to assist smokers to quit 1. Proactive telephone counselling increases cessation rates among quitline callers and actively recruited smokers (i.e. recruiter-initiated enrolment) 2,3. Smokers can be actively recruited to quitlines for proactive telephone counselling via a faxed referral from a health-care professional or smokers can call the quitline to receive reactive counselling or proactive counselling after their initial call 4.

Most (90%) US quitlines have fax-referral programmes 5. Characteristics of fax-referred smokers differ from quitline callers, highlighting that active recruitment enrols different groups of smokers 6. However, almost one-third of US quitlines offer counselling services only to smokers ready to quit within 30 days 5. Given that 80–96% of smokers do not intend to quit within 30 days 7–9, offering proactive telephone counselling based on quitting intention excludes most smokers and may result in some smokers, who could benefit, missing out on effective support.

There is a lack of proactive telephone counselling trials with actively recruited smokers that examined baseline characteristics associated with prolonged cessation. The only trial which assessed predictors of prolonged abstinence was undertaken with quitline callers 10. This study found that greater readiness to quit at baseline was associated with 3-month prolonged abstinence at 6 months, and greater telephone intervention adherence and age were associated with 6-month prolonged abstinence at 12 months 10.

Our study assessed: (i) baseline characteristics of actively recruited smokers associated with prolonged abstinence at 4, 7 or 13 months follow-up; and (ii) experimental group participants who achieved prolonged abstinence but would miss out on proactive telephone counselling if this were only offered to smokers ready to quit within 30 days.

## Methods

### Sample

Eligibility requirements were: daily tobacco use; 18 years or older; New South Wales (NSW) resident; and English-speaking.

### Procedure

Telephone numbers (*n* = 48 014) were randomly selected from the NSW electronic telephone directory. Households were mailed an information letter and telephoned. Of 43 710 households reached, 3008 contained an eligible smoker. One smoker was selected randomly, and if he/she gave verbal consent completed a baseline computer-assisted telephone interview (CATI, *n* = 1562). Subsequently, the CATI randomly allocated the smoker to proactive telephone counselling (*n* = 769) or self-help materials (*n* = 793). CATIs were conducted at 4 months (*n* = 1369), 7 months (*n* = 1278) and 13 months (*n* = 1245) to assess cessation. The detailed design and Consolidated Standards of Reporting Trials (CONSORT) diagram 11 can be found elsewhere 12. Ethics approval was granted.

### Measures

#### Baseline items

##### Socio-demographic and health items

These comprised age, gender, country of birth, Aboriginal/Torres Strait Islander, education, marital status, employment, children aged 6 years or less in household, private health insurance, area of residence, visited general practitioner in past 12 months and alcohol consumption.

##### Smoking-related items

These included time to first cigarette after waking, cigarettes smoked per day, age started smoking regularly, ever quit smoking intentionally, quit attempt in past 12 months, quitting intention, likelihood of successful quitting, other household smokers, friends/acquaintances smoke, household smoking restrictions, encouragement to quit from: family; friends; work-mates; perceived effectiveness of: you call quitline; quitline calls you; self-help materials; nicotine replacement therapy; willpower alone; and self-exempting statements.

##### Treatment condition

Assigned condition.

#### Outcome measures

Prolonged abstinence (i.e. sustained abstinence), was measured from a 1-month grace period (giving smokers opportunity to quit) to each follow-up and between interviews 13, resulting in 3, 6, 9 and 12 months’ prolonged abstinence.

### Sample size

The trial's sample size calculation indicated that 770 participants were needed per condition at 13 months to detect a 3% difference for prolonged abstinence based on a significance level of 5% and 80% power. However, for these secondary analyses, some non-significant findings may be due to limited power rather than no real difference existing. For example, in the comparison of those taking 31–60 minutes to smoke after waking with those taking 1–30 minutes the study had less than 50% power to find a doubling of the quit rate for 3-month prolonged abstinence from 2.5 to 5%.

### Statistical analysis

Statistical analysis was completed using SAS software. χ^2^ tests investigated whether baseline characteristics were associated with prolonged abstinence at 4, 7 or 13 months. Variables significant at *P* < 0.25 in the univariate analysis were included in a backward stepwise logistic regression model. Non-significant variables were removed until variables were significant at α = 0.05. Collinearity among baseline variables was not controlled for in logistic regressions because Spearman's correlation coefficient was less than 0.5 for all pairwise correlations of dichotomous and ordinal baseline variables. The pseudo *R*^2^ determined the variance accounted for in each logistic regression model and the Hosmer & Lemeshow goodness-of-fit test assessed whether the model fitted the data well.

## Results

Baseline characteristics (*n* = 1562) are described elsewhere 14.

### Predictors of prolonged abstinence

Table 1 outlines the significant predictors of prolonged abstinence at each assessment.

**Table 1:** Significant predictors of prolonged abstinence (i.e. at least 3, 6, 9 or 12 months’ abstinence) at 4, 7 and 13 months post-recruitment

	Short-term assessment	Mid-term-assessment		Long-term assessment		
			
	3-month prolonged abstinence at 4 months	3-month prolonged abstinence at 7 months	6-month prolonged abstinence at 7 months	6-month prolonged abstinence at 13 months	9-month prolonged abstinence at 13 months	12-month prolonged abstinence at 13 months
	39/1303^a^	81/1221^a^	24/1219^a^	86/1187^a^	52/1159^a^	17/1237^a^

Baseline characteristics	Odds ratio (95% CIs)	Odds ratio (95% CIs)	Odds ratio (95% CIs)	Odds ratio (95% CIs)	Odds ratio (95% CIs)	Odds ratio (95% CIs)
Marital status						
Married/*de-facto*					Referent	
Divorced/separated					0.3 (0.1–0.8)*	
Widowed					1.1 (0.2–5.2)	
Never married					0.5 (0.2–1.1)	
Employment status						
Paid employment					2.4 (1.1–5.3)*	
No paid employment					Referent	
Time to first cigarette (minutes)						
1–30	Referent	Referent	Referent	Referent		Referent
31–60	1.2 (0.5–3.1)	1.5 (0.8–2.8)	1.0 (0.3–3.5)	1.8 (1.03–3.2)*		1.1 (0.2–5.0)
61+	2.8 (1.3–6.0)*	2.2 (1.2–3.9)*	3.0 (1.2–7.8)*	2.0 (1.1–3.5)*		3.9 (1.4–11.3)*
Quitting intention						
Within 30 days	3.0 (1.3–7.3)*	2.6 (1.4–4.8)*	3.4 (1.1–10.9)*	2.4 (1.3–4.5)*	3.7 (1.5–8.7)*	
Within 6 months	1.2 (0.5–3.0)	1.2 (0.7–2.3)	1.3 (0.4–4.5)	1.7 (0.9–3.2)	2.3 (1.0–5.4)	
Not within 6 months	Referent	Referent	Referent	Referent	Referent	
Other household smokers						
Yes		1.8 (1.1–3.0)*				
No		Referent				
Friends/acquaintances smoke						
At least half				Referent		
Fewer than half				1.5 (0.9–2.5)		
None				2.5 (1.1–5.4)*		
Perceived effectiveness of willpower alone						
Not at all effective		Referent		Referent		
Partly effective		0.5 (0.3–0.98)*		0.6 (0.3–0.99)*		
Definitely effective		1.4 (0.8–2.4)		1.4(0.8–2.4)		
Don't know		1.1 (0.1–8.7)		0.9 (0.1–7.3)		
Alcohol consumption						
Don't drink alcohol	0.7 (0.3–1.6)		0.8 (0.3–2.4)			1.0 (0.3–3.7)
Daily	0.5 (0.2–1.3)		0.6 (0.2–1.9)			1.1 (0.3–3.7)
Weekly	0.3 (0.1–0.6)*		0.2 (0.1–0.6)*			0.2 (0.03–0.8)*
Less than weekly	Referent		Referent			Referent
Treatment condition						
Proactive telephone counselling			2.6 (1.1–6.3)*			
Control			Referent			
Pseudo *R*^2^	0.07	0.06	0.10	0.05	0.06	0.08
Hosmer & Lemeshow goodness-of-fit test (*P*-value)^b^	0.09	0.8	0.6	0.3	0.9	0.8

a Based on the observations used by the logistic regression. Observations deleted due to missing values are not included in the numerator or denominator.

b The Hosmer & Lemeshow goodness-of-fit test suggested that each backward stepwise logistic regression model fitted the data well. CI: confidence interval.

* *P* < 0.05.

#### Predictors of prolonged abstinence in the short term

##### Three-month prolonged abstinence at 4 months

Participants who, at baseline, smoked more than an hour after waking or intended to quit within 30 days had greater odds of 3-month prolonged abstinence at 4 months. Weekly alcohol drinkers had smaller odds of abstinence.

#### Predictors of prolonged abstinence in the mid-term

##### Three-month prolonged abstinence at 7 months

Those who, at baseline, smoked more than an hour after waking, intended to quit within 30 days or lived with other smokers had larger odds of 3-month prolonged abstinence at 7 months. Participants who perceived willpower alone as partly effective for quitting had smaller odds of abstinence.

##### Six-month prolonged abstinence at 7 months

Participants who, at baseline, smoked more than an hour after waking, intended to quit within 30 days or were offered telephone counselling had greater odds of 6-month prolonged abstinence at 7 months. Weekly alcohol drinkers had smaller odds of abstinence.

#### Predictors of prolonged abstinence in the long term

##### Six-month prolonged abstinence at 13 months

Participants who, at baseline, waited 31 or more minutes after waking to smoke, intended to quit within 30 days or none of their friends/acquaintances smoked had greater odds of 6-month prolonged abstinence at 13 months. Those who perceived willpower alone as partly effective for quitting had smaller odds of abstinence.

##### Nine-month prolonged abstinence at 13 months

Divorced/separated smokers had smaller odds of 9-month prolonged abstinence at 13 months. Employed participants and those intending to quit within 30 days had larger odds of abstinence.

##### Twelve-month prolonged abstinence at 13 months

Participants who, at baseline, smoked more than an hour after waking had greater odds of 12-month prolonged abstinence at 13 months, whereas smokers who consumed alcohol weekly had smaller odds.

#### Consistent predictors of prolonged abstinence

Waiting more than an hour to smoke after waking and intention to quit within 30 days were significant predictors on five of the six prolonged abstinence measures.

#### Restricting proactive telephone counselling to smokers intending to quit within 30 days

Experimental group participants who achieved prolonged abstinence but would miss out on proactive telephone counselling if such support were offered only to smokers who at baseline intended to quit within 30 days would be: 3-month prolonged abstinence: 14/26 (53.8%) at 4 months, 26/46 (56.5%) at 7 months; 6-month prolonged abstinence: 10/17 (58.8%) at 7 months, 29/44 (65.9%) at 13 months; 9-month prolonged abstinence: 15/25 (60.0%) at 13 months; and 12-month prolonged abstinence: 7/11 (63.6%) at 13 months.

## Discussion

This research found that waiting more than an hour to smoke after waking and intending to quit within 30 days most consistently predicted prolonged abstinence among smokers actively recruited to quitline support. We also found that if proactive telephone counselling were offered only to smokers who intended to quit within 30 days, then 53.8–65.9% of experimental group participants who achieved prolonged abstinence would miss out on telephone support.

The study strengths included that all smokers irrespective of quitting intention were eligible and retention rates at follow-ups were high. These features increase the generalizability of the findings to the general smoking population. Study shortcomings included that limited power for secondary analyses may have contributed to non-significant findings for some baseline characteristics. Furthermore, the reliability of the percentages of quitters who would miss out on proactive telephone counselling if support were offered only to those intending to quit within 30 days was limited by small samples. Biochemical validation of self-reported cessation was not conducted; however, it is considered unnecessary for such trials 15.

Our 7-month findings are similar to a trial with quitline callers that reported that greater readiness to quit was associated with 3-month prolonged abstinence at 6 months 10. However, unlike the prior study, we found that intention to quit also predicted prolonged abstinence longer-term. A cohort study of smokers recruited from community health centres into proactive telephone support found that less addiction predicted prolonged abstinence at 30 days 16. We found an association between nicotine dependence and prolonged abstinence also existed at 4, 7 and 13 months.

Longer time to first cigarette after waking (a validated measure of nicotine dependence 17) was critical in determining whether actively recruited smokers achieved prolonged abstinence. Quitline advisers should therefore assess actively recruited smokers’ nicotine dependence and tailor advice accordingly; for example, by encouraging use of pharmacotherapies 18. Despite more than 70% of actively recruited smokers reporting no intention to quit within the next 30 days at baseline 12, such smokers still achieved long-term abstinence.
